# Crystal structure of a second polymorph of 2-cyclo­penta­dienyl-1,7-dicarba-2-cobalta-*closo*-dodeca­borane(11)

**DOI:** 10.1107/S2056989015011445

**Published:** 2015-06-17

**Authors:** Wing Y. Man, Georgina M. Rosair, Alan J. Welch

**Affiliations:** a Institute of Chemical Sciences, School of Engineering & Physical Sciences, Heriot-Watt University, Edinburgh EH14 4AS, Scotland

**Keywords:** metallacarborane, polymorph, crystal structure

## Abstract

A new polymorph of the title compound 2-(η-C_5_H_5_)-2,1,7-*closo*-CoC_2_B_9_H_11_, [Co(C_5_H_5_)(C_2_H_11_B_9_)], in the space group *P*2_1_/*n* has been characterized, including the unambiguous location of both cage C atoms. The precision of this study is an order of magnitude greater than that of the first polymorph [*C*2/*c*; Lopez *et al.* (2010). *Collect. Czech. Chem. Commun*. **75**, 853–869].

## Related literature   

For the structure of the *C*2/*c* polymorph, see: Lopez *et al.* (2010 ▸). For structures of other (η-C_5_H_5_)CoC_2_B_9_H_11_ isomers, see: Smith & Welch (1986 ▸), Lopez *et al.* (2010 ▸) and Man *et al.* (2014 ▸). Methods used to identify cage C atoms: *Vertex-to-Centroid Distance* (McAnaw *et al.*, 2013 ▸) and *Boron-Hydrogen Distance* (McAnaw *et al.*, 2014 ▸).
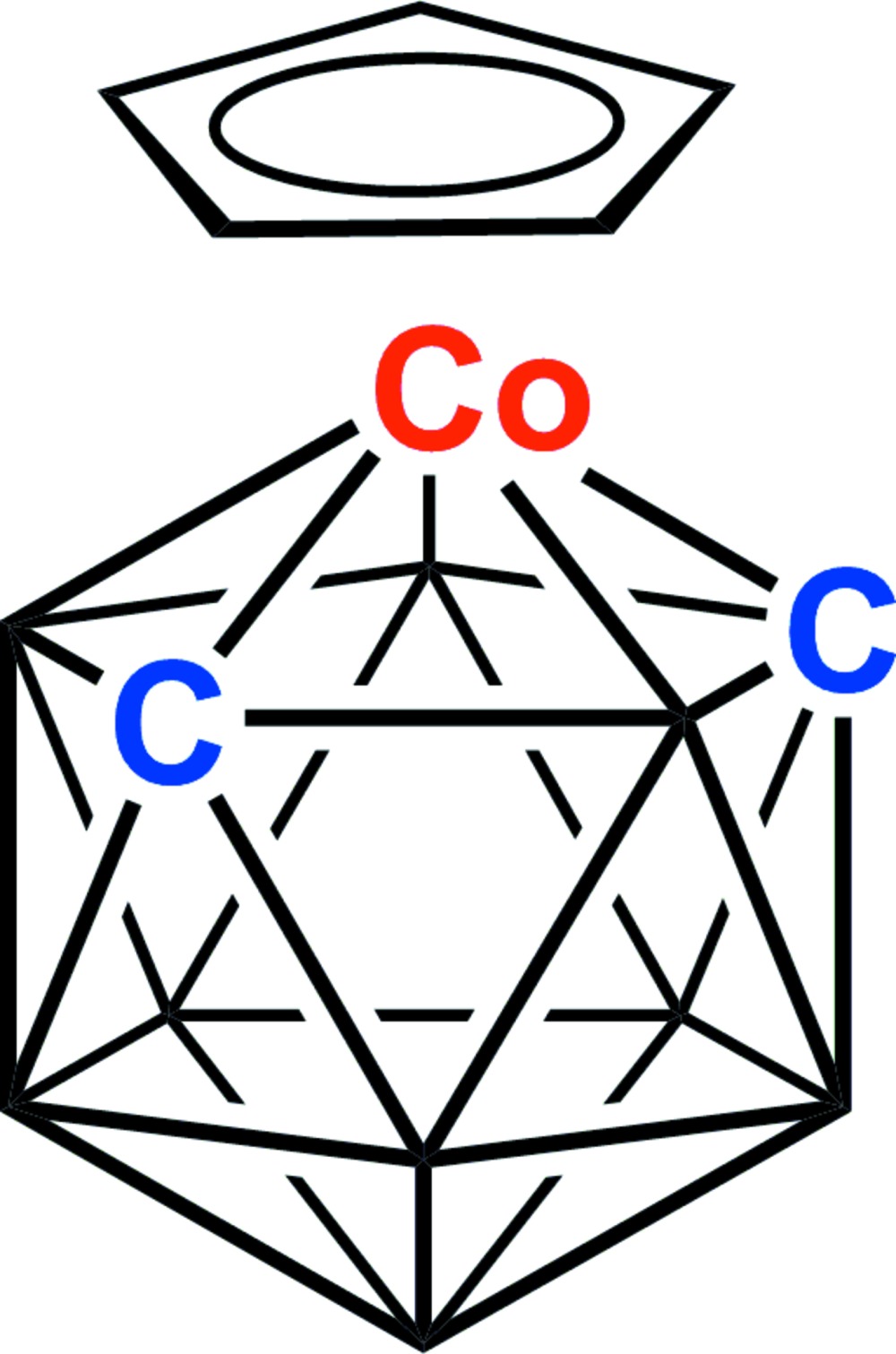


## Experimental   

### Crystal data  

[Co(C_5_H_5_)(C_2_H_11_B_9_)]
*M* *_r_* = 256.42
Monoclinic, 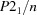

*a* = 12.4903 (11) Å
*b* = 8.7207 (7) Å
*c* = 12.7392 (12) Å
β = 116.123 (4)°
*V* = 1245.86 (19) Å^3^
*Z* = 4
Mo *K*α radiation
μ = 1.34 mm^−1^
*T* = 100 K
0.46 × 0.44 × 0.16 mm

### Data collection  

Bruker APEXII CCD diffractometer
Absorption correction: multi-scan (*SADABS*; Bruker,2008 ▸) *T*_min_ = 0.649, *T*_max_ = 0.747
37238 measured reflections
5019 independent reflections
4268 reflections with *I* > 2σ(*I*)
*R*_int_ = 0.033

### Refinement  

*R*[*F* ^2^ > 2σ(*F* ^2^)] = 0.022
*wR*(*F* ^2^) = 0.058
*S* = 1.03
5019 reflections
218 parameters
All H-atom parameters refined
Δρ_max_ = 0.41 e Å^−3^
Δρ_min_ = −0.41 e Å^−3^

### 

Data collection: *APEX2* (Bruker, 2011 ▸); cell refinement: *SAINT* (Bruker, 2011 ▸); data reduction: *SAINT*; program(s) used to solve structure: *SHELXS97* (Sheldrick, 2008 ▸); program(s) used to refine structure: *SHELXL2014* (Sheldrick, 2015 ▸); molecular graphics: *OLEX2* (Dolomanov *et al.*, 2009 ▸); software used to prepare material for publication: *OLEX2*.

## Supplementary Material

Crystal structure: contains datablock(s) I. DOI: 10.1107/S2056989015011445/pj2020sup1.cif

Structure factors: contains datablock(s) I. DOI: 10.1107/S2056989015011445/pj2020Isup2.hkl

Supporting information file. DOI: 10.1107/S2056989015011445/pj2020Isup3.mol

**Fig. 1.**
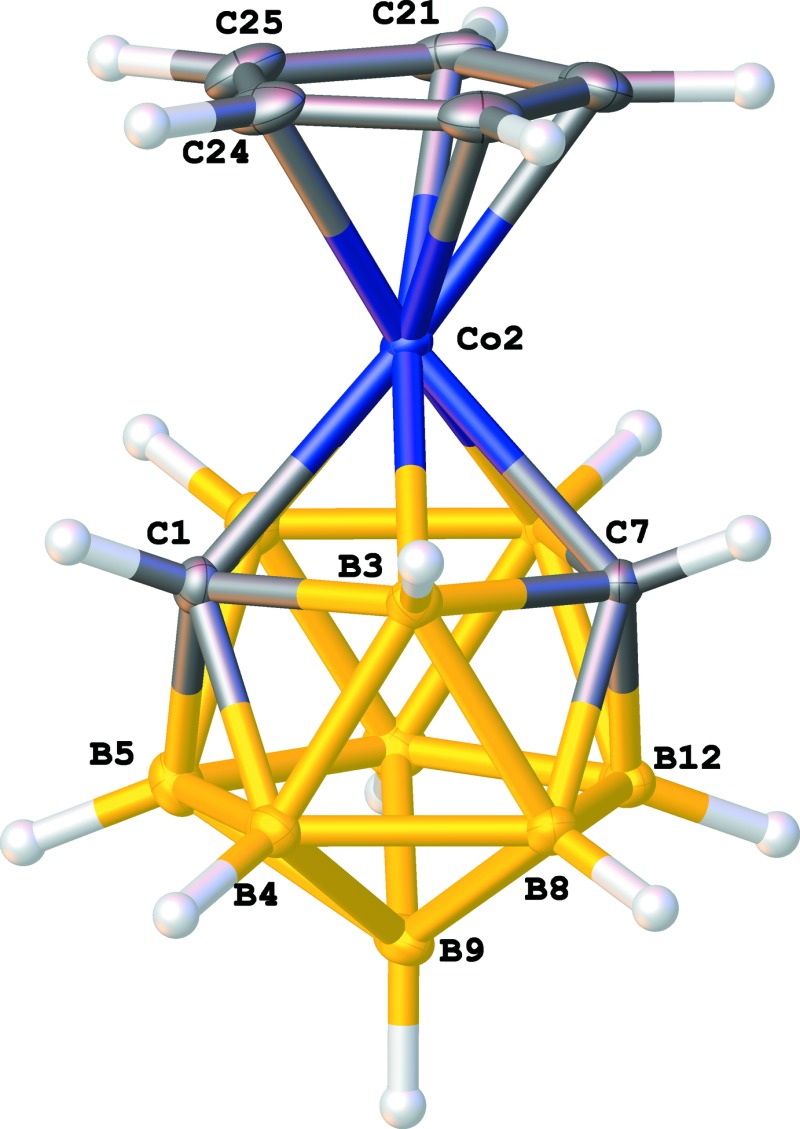
Perspective view of the title compound with atom numbering and displacement ellipsoids drawn at the 50% probability level except for H atoms.

. DOI: 10.1107/S2056989015011445/pj2020fig1.tif
Perspective view of the title compound with atom numbering and displacement ellipsoids drawn at the 50% probability level except for H atoms.

**Fig. 2.**
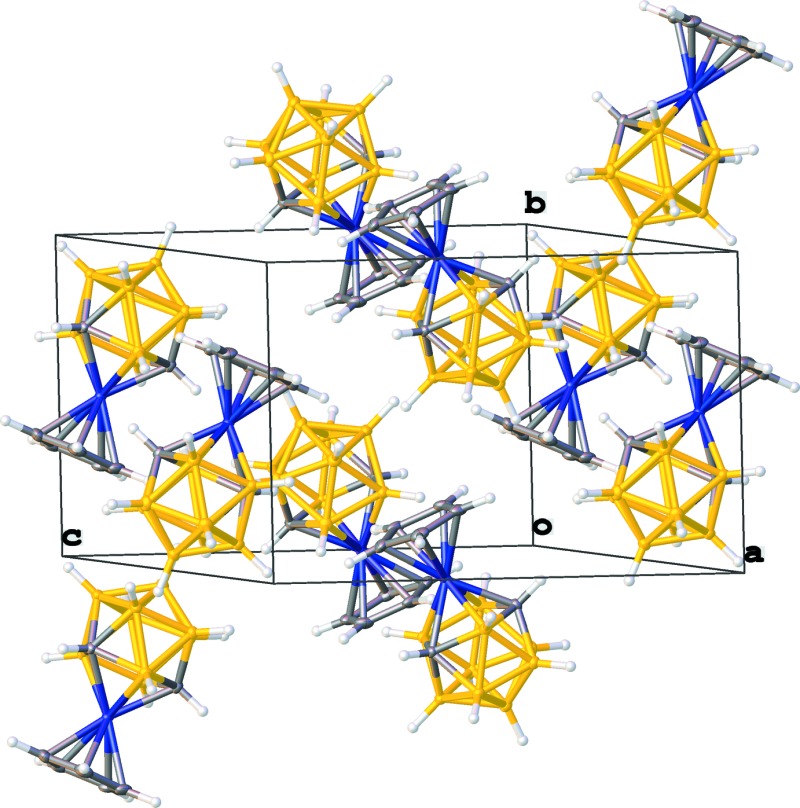
Packing diagram of the title compound.

. DOI: 10.1107/S2056989015011445/pj2020fig2.tif
Packing diagram of the title compound.

CCDC reference: 1406489

Additional supporting information:  crystallographic information; 3D view; checkCIF report

## Figures and Tables

**Table 1:** Selected bond lengths ()

C1Co2	2.0556(8)
Co2B3	2.0471(9)
Co2B6	2.0762(9)
Co2C7	2.0539(8)
Co2B11	2.0746(10)
Co2C21	2.0574(9)
Co2C22	2.0536(9)
Co2C23	2.0759(9)
Co2C24	2.0823(9)
Co2C25	2.0548(9)

## supplementary crystallographic information

### S1. Synthesis and crystallization

The compound was prepared as previously reported (Lopez *et al.* 2010) and purity established by NMR spectroscopy: ^1^H NMR (CDCl_3_); 5.64 (s, 5 H, C_5_H_5_), 2.65 (br s, 2 H, CH_cage_). ^11^B{^1^H} NMR (CDCl_3_): 2.9 (1 B), -2.4 (2 B), -9.0 (1 B), -10.8 (1 B), -11.8 (2 B), -17.0 (2 B). Single crystals were grown by diffusion of a solution of the compound in CH_2_Cl_2_ and petroleum ether at 4 °C.

### S2. Refinement

Crystal data, data collection and structure refinement details are summarized in Table 1. The carbon atoms in the metallaccarborane were located using the Vertex-to-Centroid Distance (VCD) (McAnaw *et al.*, 2013) and Boron-Hydrogen Distance (BHD) (McAnaw *et al.*, 2014) methods that we have developed for C/B discrimination. In this case the methods afford unequivocal assignment of the carbon location.

### Crystal data

**Table:**

[Co(C_5_H_5_)(C_2_H_11_B_9_)]	*F*(000) = 520
*M**_r_* = 256.42	*D*_x_ = 1.367 Mg m^−^^3^
Monoclinic, *P*2_1_/*n*	Mo *K*α radiation, λ = 0.71073 Å
*a* = 12.4903 (11) Å	Cell parameters from 9899 reflections
*b* = 8.7207 (7) Å	θ = 2.9–33.8°
*c* = 12.7392 (12) Å	µ = 1.34 mm^−^^1^
β = 116.123 (4)°	*T* = 100 K
*V* = 1245.86 (19) Å^3^	BLOCK, yellow
*Z* = 4	0.46 × 0.44 × 0.16 mm

### Data collection

**Table:**

Bruker APEXII CCD diffractometer	4268 reflections with *I* > 2σ(*I*)
Graphite monochromator	*R*_int_ = 0.033
φ and ω scans	θ_max_ = 33.9°, θ_min_ = 2.9°
Absorption correction: multi-scan (*SADABS*; Bruker,2008)	*h* = −19→19
*T*_min_ = 0.649, *T*_max_ = 0.747	*k* = −13→13
37238 measured reflections	*l* = −19→19
5019 independent reflections	

### Refinement

**Table:**

Refinement on *F*^2^	Primary atom site location: iterative
Least-squares matrix: full	Hydrogen site location: difference Fourier map
*R*[*F*^2^ > 2σ(*F*^2^)] = 0.022	All H-atom parameters refined
*wR*(*F*^2^) = 0.058	*w* = 1/[σ^2^(*F*_o_^2^) + (0.0318*P*)^2^ + 0.1768*P*] where *P* = (*F*_o_^2^ + 2*F*_c_^2^)/3
*S* = 1.03	(Δ/σ)_max_ < 0.001
5019 reflections	Δρ_max_ = 0.41 e Å^−^^3^
218 parameters	Δρ_min_ = −0.41 e Å^−^^3^
0 restraints	

### Special details

**Table:**

Geometry. All e.s.d.'s (except the e.s.d. in the dihedral angle between two l.s. planes) are estimated using the full covariance matrix. The cell e.s.d.'s are taken into account individually in the estimation of e.s.d.'s in distances, angles and torsion angles; correlations between e.s.d.'s in cell parameters are only used when they are defined by crystal symmetry. An approximate (isotropic) treatment of cell e.s.d.'s is used for estimating e.s.d.'s involving l.s. planes.

### Fractional atomic coordinates and isotropic or equivalent isotropic displacement parameters (Å^2^)

**Table:**

	*x*	*y*	*z*	*U*_iso_*/*U*_eq_	
C1	0.18879 (7)	0.25685 (9)	0.41663 (7)	0.01285 (13)	
H1	0.1431 (10)	0.2529 (14)	0.3377 (10)	0.017 (3)*	
Co2	0.23995 (2)	0.47776 (2)	0.47249 (2)	0.01116 (4)	
B3	0.13135 (8)	0.33118 (11)	0.50692 (8)	0.01316 (15)	
H3	0.0422 (11)	0.3767 (15)	0.4780 (11)	0.024 (3)*	
B4	0.15056 (9)	0.12828 (11)	0.49708 (8)	0.01494 (16)	
H4	0.0737 (11)	0.0584 (14)	0.4513 (11)	0.022 (3)*	
B5	0.28116 (9)	0.10264 (11)	0.47514 (8)	0.01595 (17)	
H5	0.2862 (13)	0.0131 (15)	0.4169 (14)	0.027 (4)*	
B6	0.33933 (9)	0.28936 (11)	0.46770 (8)	0.01428 (16)	
H6	0.3824 (11)	0.3037 (15)	0.4101 (11)	0.024 (3)*	
C7	0.25585 (7)	0.39021 (10)	0.62837 (7)	0.01336 (14)	
H7	0.2472 (12)	0.4668 (14)	0.6773 (12)	0.021 (3)*	
B8	0.19485 (9)	0.21634 (11)	0.63631 (8)	0.01462 (16)	
H8	0.1486 (11)	0.2066 (15)	0.6925 (11)	0.025 (3)*	
B9	0.28960 (9)	0.07601 (12)	0.61704 (8)	0.01597 (16)	
H9	0.3044 (13)	−0.0368 (16)	0.6573 (13)	0.029 (4)*	
B10	0.40638 (9)	0.17746 (12)	0.59941 (9)	0.01720 (17)	
H10	0.4968 (12)	0.1298 (16)	0.6298 (11)	0.029 (3)*	
B11	0.38440 (8)	0.37968 (12)	0.61129 (8)	0.01579 (16)	
H11	0.4607 (12)	0.4570 (15)	0.6573 (13)	0.024 (3)*	
B12	0.35215 (9)	0.24439 (12)	0.70026 (8)	0.01705 (17)	
H12	0.4026 (12)	0.2480 (16)	0.7955 (12)	0.033 (4)*	
C21	0.31179 (9)	0.63450 (11)	0.40069 (10)	0.02336 (19)	
H21	0.3802 (13)	0.6185 (17)	0.3948 (13)	0.036 (4)*	
C22	0.30148 (9)	0.69955 (11)	0.49850 (10)	0.02242 (18)	
H22	0.3669 (12)	0.7371 (17)	0.5696 (13)	0.035 (4)*	
C23	0.17796 (9)	0.69813 (10)	0.47434 (9)	0.02153 (18)	
H23	0.1474 (12)	0.7323 (17)	0.5280 (13)	0.036 (4)*	
C24	0.11213 (9)	0.63137 (11)	0.36254 (9)	0.02209 (18)	
H24	0.0275 (12)	0.6137 (16)	0.3250 (12)	0.032 (4)*	
C25	0.19471 (10)	0.59072 (11)	0.31712 (9)	0.02345 (19)	
H25	0.1756 (13)	0.5399 (16)	0.2449 (14)	0.031 (4)*	

### Atomic displacement parameters (Å^2^)

**Table:**

	*U*^11^	*U*^22^	*U*^33^	*U*^12^	*U*^13^	*U*^23^
C1	0.0165 (3)	0.0112 (3)	0.0104 (3)	−0.0015 (3)	0.0055 (3)	−0.0007 (2)
Co2	0.01304 (5)	0.00859 (5)	0.01386 (5)	−0.00067 (3)	0.00776 (4)	−0.00026 (4)
B3	0.0129 (4)	0.0129 (4)	0.0144 (4)	−0.0013 (3)	0.0067 (3)	−0.0001 (3)
B4	0.0190 (4)	0.0118 (4)	0.0148 (4)	−0.0026 (3)	0.0081 (3)	0.0003 (3)
B5	0.0236 (4)	0.0117 (4)	0.0161 (4)	0.0014 (3)	0.0120 (3)	0.0010 (3)
B6	0.0173 (4)	0.0127 (4)	0.0164 (4)	0.0015 (3)	0.0107 (3)	0.0005 (3)
C7	0.0151 (3)	0.0136 (3)	0.0125 (3)	−0.0008 (3)	0.0071 (3)	−0.0025 (3)
B8	0.0183 (4)	0.0142 (4)	0.0139 (4)	0.0001 (3)	0.0093 (3)	0.0010 (3)
B9	0.0207 (4)	0.0142 (4)	0.0148 (4)	0.0027 (3)	0.0095 (3)	0.0031 (3)
B10	0.0164 (4)	0.0178 (4)	0.0185 (4)	0.0038 (3)	0.0086 (3)	0.0028 (3)
B11	0.0135 (4)	0.0177 (4)	0.0161 (4)	−0.0010 (3)	0.0065 (3)	−0.0013 (3)
B12	0.0172 (4)	0.0202 (4)	0.0125 (4)	0.0012 (3)	0.0054 (3)	0.0005 (3)
C21	0.0284 (5)	0.0134 (4)	0.0401 (5)	0.0008 (3)	0.0258 (4)	0.0056 (4)
C22	0.0237 (4)	0.0110 (4)	0.0331 (5)	−0.0036 (3)	0.0131 (4)	−0.0013 (3)
C23	0.0289 (5)	0.0104 (4)	0.0340 (5)	0.0031 (3)	0.0217 (4)	0.0020 (3)
C24	0.0216 (4)	0.0155 (4)	0.0287 (5)	0.0037 (3)	0.0107 (4)	0.0087 (3)
C25	0.0375 (5)	0.0159 (4)	0.0218 (4)	0.0023 (4)	0.0175 (4)	0.0064 (3)

### Geometric parameters (Å, º)

**Table:**

C1—H1	0.912 (12)	B6—B10	1.7976 (14)
C1—Co2	2.0556 (8)	B6—B11	1.8376 (13)
C1—B3	1.7276 (12)	C7—H7	0.952 (14)
C1—B4	1.7224 (12)	C7—B8	1.7198 (13)
C1—B5	1.7141 (13)	C7—B11	1.7150 (13)
C1—B6	1.7210 (13)	C7—B12	1.7125 (13)
Co2—B3	2.0471 (9)	B8—H8	1.104 (13)
Co2—B6	2.0762 (9)	B8—B9	1.7928 (14)
Co2—C7	2.0539 (8)	B8—B12	1.7815 (14)
Co2—B11	2.0746 (10)	B9—H9	1.087 (14)
Co2—C21	2.0574 (9)	B9—B10	1.8025 (14)
Co2—C22	2.0536 (9)	B9—B12	1.7780 (15)
Co2—C23	2.0759 (9)	B10—H10	1.101 (13)
Co2—C24	2.0823 (9)	B10—B11	1.8014 (14)
Co2—C25	2.0548 (9)	B10—B12	1.7935 (14)
B3—H3	1.082 (12)	B11—H11	1.102 (13)
B3—B4	1.7974 (13)	B11—B12	1.8002 (14)
B3—C7	1.7199 (12)	B12—H12	1.094 (14)
B3—B8	1.7882 (13)	C21—H21	0.901 (14)
B4—H4	1.068 (13)	C21—C22	1.4256 (15)
B4—B5	1.7863 (14)	C21—C25	1.4306 (15)
B4—B8	1.7825 (13)	C22—H22	0.971 (15)
B4—B9	1.7950 (14)	C22—C23	1.4343 (14)
B5—H5	1.098 (14)	C23—H23	0.966 (15)
B5—B6	1.8025 (14)	C23—C24	1.4185 (15)
B5—B9	1.7790 (14)	C24—H24	0.961 (14)
B5—B10	1.7882 (14)	C24—C25	1.4319 (14)
B6—H6	1.091 (13)	C25—H25	0.952 (15)
			
Co2—C1—H1	111.4 (8)	Co2—C7—H7	112.4 (8)
B3—C1—H1	120.4 (7)	B3—C7—Co2	65.00 (4)
B3—C1—Co2	64.84 (4)	B3—C7—H7	118.7 (8)
B4—C1—H1	115.3 (8)	B8—C7—Co2	121.86 (5)
B4—C1—Co2	121.97 (5)	B8—C7—B3	62.65 (5)
B4—C1—B3	62.79 (5)	B8—C7—H7	113.5 (8)
B5—C1—H1	115.1 (8)	B11—C7—Co2	66.09 (4)
B5—C1—Co2	122.52 (6)	B11—C7—B3	113.33 (6)
B5—C1—B3	113.71 (6)	B11—C7—H7	120.2 (8)
B5—C1—B4	62.64 (5)	B11—C7—B8	115.09 (7)
B5—C1—B6	63.30 (6)	B12—C7—Co2	122.64 (5)
B6—C1—H1	118.2 (7)	B12—C7—B3	113.75 (7)
B6—C1—Co2	66.01 (4)	B12—C7—H7	115.3 (8)
B6—C1—B3	113.04 (6)	B12—C7—B8	62.54 (6)
B6—C1—B4	115.17 (6)	B12—C7—B11	63.37 (6)
C1—Co2—B6	49.23 (4)	B3—B8—H8	119.6 (7)
C1—Co2—B11	85.97 (4)	B3—B8—B9	107.92 (6)
C1—Co2—C21	126.78 (4)	B4—B8—B3	60.44 (5)
C1—Co2—C23	144.18 (4)	B4—B8—H8	126.2 (7)
C1—Co2—C24	109.92 (4)	B4—B8—B9	60.27 (5)
B3—Co2—C1	49.81 (3)	C7—B8—B3	58.68 (5)
B3—Co2—B6	88.47 (4)	C7—B8—B4	105.83 (6)
B3—Co2—C7	49.59 (3)	C7—B8—H8	118.5 (7)
B3—Co2—B11	88.25 (4)	C7—B8—B9	104.92 (7)
B3—Co2—C21	165.93 (4)	C7—B8—B12	58.53 (5)
B3—Co2—C22	142.17 (4)	B9—B8—H8	127.0 (7)
B3—Co2—C23	106.86 (4)	B12—B8—B3	107.27 (7)
B3—Co2—C24	99.31 (4)	B12—B8—B4	108.11 (7)
B3—Co2—C25	125.36 (4)	B12—B8—H8	120.0 (7)
B6—Co2—C24	141.31 (4)	B12—B8—B9	59.66 (6)
C7—Co2—C1	82.49 (3)	B4—B9—H9	122.1 (8)
C7—Co2—B6	85.97 (3)	B4—B9—B10	107.58 (7)
C7—Co2—B11	49.09 (4)	B5—B9—B4	59.97 (5)
C7—Co2—C21	143.26 (4)	B5—B9—B8	107.62 (7)
C7—Co2—C23	102.18 (4)	B5—B9—H9	121.3 (8)
C7—Co2—C24	127.49 (4)	B5—B9—B10	59.90 (6)
C7—Co2—C25	167.97 (4)	B8—B9—B4	59.58 (5)
B11—Co2—B6	52.55 (4)	B8—B9—H9	122.6 (8)
B11—Co2—C23	124.11 (4)	B8—B9—B10	107.53 (7)
B11—Co2—C24	163.74 (4)	B10—B9—H9	121.6 (7)
C21—Co2—B6	97.43 (4)	B12—B9—B4	107.71 (7)
C21—Co2—B11	105.49 (4)	B12—B9—B5	108.18 (7)
C21—Co2—C23	68.15 (4)	B12—B9—B8	59.86 (6)
C21—Co2—C24	68.21 (4)	B12—B9—H9	121.8 (8)
C22—Co2—C1	167.36 (4)	B12—B9—B10	60.12 (6)
C22—Co2—B6	124.33 (4)	B5—B10—B6	60.35 (5)
C22—Co2—C7	108.91 (4)	B5—B10—B9	59.40 (5)
C22—Co2—B11	97.33 (4)	B5—B10—H10	122.4 (7)
C22—Co2—C21	40.58 (4)	B5—B10—B11	108.99 (7)
C22—Co2—C23	40.64 (4)	B5—B10—B12	107.09 (7)
C22—Co2—C24	67.93 (4)	B6—B10—B9	108.22 (7)
C22—Co2—C25	68.20 (4)	B6—B10—H10	121.2 (7)
C23—Co2—B6	164.52 (4)	B6—B10—B11	61.40 (5)
C23—Co2—C24	39.89 (4)	B9—B10—H10	122.1 (7)
C25—Co2—C1	101.78 (4)	B11—B10—B9	107.96 (7)
C25—Co2—B6	105.39 (4)	B11—B10—H10	120.7 (7)
C25—Co2—B11	141.79 (4)	B12—B10—B6	108.92 (7)
C25—Co2—C21	40.72 (4)	B12—B10—B9	59.26 (6)
C25—Co2—C23	67.75 (4)	B12—B10—H10	121.5 (7)
C25—Co2—C24	40.49 (4)	B12—B10—B11	60.10 (6)
C1—B3—Co2	65.35 (4)	Co2—B11—H11	115.1 (7)
C1—B3—H3	125.2 (7)	B6—B11—Co2	63.77 (4)
C1—B3—B4	58.46 (5)	B6—B11—H11	127.3 (7)
C1—B3—B8	104.94 (6)	C7—B11—Co2	64.83 (4)
Co2—B3—H3	112.1 (7)	C7—B11—B6	104.81 (6)
B4—B3—Co2	118.58 (6)	C7—B11—B10	104.42 (7)
B4—B3—H3	119.1 (7)	C7—B11—H11	122.6 (7)
C7—B3—C1	103.60 (6)	C7—B11—B12	58.25 (5)
C7—B3—Co2	65.41 (4)	B10—B11—Co2	116.07 (6)
C7—B3—H3	125.7 (7)	B10—B11—B6	59.20 (5)
C7—B3—B4	105.18 (6)	B10—B11—H11	121.0 (7)
C7—B3—B8	58.67 (5)	B12—B11—Co2	117.07 (6)
B8—B3—Co2	118.71 (6)	B12—B11—B6	106.88 (7)
B8—B3—H3	119.5 (7)	B12—B11—B10	59.73 (6)
B8—B3—B4	59.62 (5)	B12—B11—H11	116.5 (7)
C1—B4—B3	58.75 (5)	C7—B12—B8	58.93 (5)
C1—B4—H4	117.7 (7)	C7—B12—B9	105.88 (7)
C1—B4—B5	58.45 (5)	C7—B12—B10	104.86 (7)
C1—B4—B8	105.41 (6)	C7—B12—B11	58.39 (5)
C1—B4—B9	104.72 (6)	C7—B12—H12	119.8 (7)
B3—B4—H4	119.0 (7)	B8—B12—B10	108.42 (7)
B5—B4—B3	107.06 (6)	B8—B12—B11	108.03 (7)
B5—B4—H4	120.4 (7)	B8—B12—H12	119.5 (7)
B5—B4—B9	59.57 (5)	B9—B12—B8	60.49 (5)
B8—B4—B3	59.93 (5)	B9—B12—B10	60.62 (6)
B8—B4—H4	126.8 (7)	B9—B12—B11	109.10 (7)
B8—B4—B5	107.75 (7)	B9—B12—H12	124.9 (8)
B8—B4—B9	60.15 (5)	B10—B12—B11	60.17 (6)
B9—B4—B3	107.43 (7)	B10—B12—H12	126.1 (7)
B9—B4—H4	128.3 (7)	B11—B12—H12	120.0 (7)
C1—B5—B4	58.91 (5)	Co2—C21—H21	123.4 (9)
C1—B5—H5	119.5 (8)	C22—C21—Co2	69.57 (5)
C1—B5—B6	58.54 (5)	C22—C21—H21	126.1 (9)
C1—B5—B9	105.77 (7)	C22—C21—C25	107.49 (9)
C1—B5—B10	104.93 (7)	C25—C21—Co2	69.55 (5)
B4—B5—H5	121.5 (8)	C25—C21—H21	126.3 (9)
B4—B5—B6	108.19 (6)	Co2—C22—H22	124.7 (9)
B4—B5—B10	108.59 (7)	C21—C22—Co2	69.85 (5)
B6—B5—H5	117.6 (8)	C21—C22—H22	125.9 (8)
B9—B5—B4	60.46 (5)	C21—C22—C23	108.15 (9)
B9—B5—H5	126.8 (8)	C23—C22—Co2	70.51 (5)
B9—B5—B6	109.05 (7)	C23—C22—H22	125.9 (8)
B9—B5—B10	60.70 (6)	Co2—C23—H23	124.0 (9)
B10—B5—H5	124.7 (8)	C22—C23—Co2	68.84 (5)
B10—B5—B6	60.08 (5)	C22—C23—H23	124.7 (8)
C1—B6—Co2	64.76 (4)	C24—C23—Co2	70.30 (5)
C1—B6—B5	58.16 (5)	C24—C23—C22	108.20 (9)
C1—B6—H6	123.0 (7)	C24—C23—H23	127.1 (8)
C1—B6—B10	104.23 (6)	Co2—C24—H24	125.5 (8)
C1—B6—B11	104.58 (6)	C23—C24—Co2	69.81 (5)
Co2—B6—H6	114.0 (7)	C23—C24—H24	126.1 (8)
B5—B6—Co2	116.96 (6)	C23—C24—C25	107.76 (9)
B5—B6—H6	117.9 (7)	C25—C24—Co2	68.72 (5)
B5—B6—B11	106.78 (6)	C25—C24—H24	126.1 (8)
B10—B6—Co2	116.17 (6)	Co2—C25—H25	123.6 (9)
B10—B6—B5	59.56 (5)	C21—C25—Co2	69.74 (6)
B10—B6—H6	121.7 (7)	C21—C25—C24	108.38 (9)
B10—B6—B11	59.40 (6)	C21—C25—H25	125.6 (9)
B11—B6—Co2	63.68 (4)	C24—C25—Co2	70.79 (5)
B11—B6—H6	126.5 (7)	C24—C25—H25	126.0 (9)
			
C1—B3—B4—B5	34.27 (6)	B5—C1—B4—B9	39.12 (6)
C1—B3—B4—B8	135.28 (7)	B5—C1—B6—Co2	−151.95 (5)
C1—B3—B4—B9	96.95 (7)	B5—C1—B6—B10	−39.40 (6)
C1—B3—C7—Co2	54.37 (5)	B5—C1—B6—B11	−100.94 (7)
C1—B3—C7—B8	−99.40 (7)	B5—B4—B8—B3	99.82 (7)
C1—B3—C7—B11	7.86 (9)	B5—B4—B8—C7	61.25 (8)
C1—B3—C7—B12	−62.05 (8)	B5—B4—B8—B9	−37.17 (6)
C1—B3—B8—B4	−38.37 (6)	B5—B4—B8—B12	−0.18 (9)
C1—B3—B8—C7	97.03 (6)	B5—B4—B9—B8	138.14 (7)
C1—B3—B8—B9	0.14 (8)	B5—B4—B9—B10	37.75 (6)
C1—B3—B8—B12	63.05 (8)	B5—B4—B9—B12	101.17 (7)
C1—B4—B5—B6	32.89 (6)	B5—B6—B10—B9	−36.47 (6)
C1—B4—B5—B9	134.94 (7)	B5—B6—B10—B11	−137.44 (7)
C1—B4—B5—B10	96.58 (7)	B5—B6—B10—B12	−99.35 (8)
C1—B4—B8—B3	38.61 (6)	B5—B6—B11—Co2	−112.27 (6)
C1—B4—B8—C7	0.04 (8)	B5—B6—B11—C7	−60.65 (8)
C1—B4—B8—B9	−98.38 (7)	B5—B6—B11—B10	37.52 (6)
C1—B4—B8—B12	−61.39 (8)	B5—B6—B11—B12	0.10 (9)
C1—B4—B9—B5	−38.58 (6)	B5—B9—B10—B6	36.89 (6)
C1—B4—B9—B8	99.56 (7)	B5—B9—B10—B11	101.87 (7)
C1—B4—B9—B10	−0.84 (8)	B5—B9—B10—B12	138.51 (7)
C1—B4—B9—B12	62.58 (8)	B5—B9—B12—C7	61.28 (8)
C1—B5—B6—Co2	28.50 (5)	B5—B9—B12—B8	100.24 (7)
C1—B5—B6—B10	134.47 (7)	B5—B9—B12—B10	−37.11 (7)
C1—B5—B6—B11	97.02 (7)	B5—B9—B12—B11	−0.15 (9)
C1—B5—B9—B4	39.04 (6)	B5—B10—B11—Co2	−8.27 (9)
C1—B5—B9—B8	1.90 (9)	B5—B10—B11—B6	−38.43 (6)
C1—B5—B9—B10	−98.54 (7)	B5—B10—B11—C7	60.43 (8)
C1—B5—B9—B12	−61.34 (8)	B5—B10—B11—B12	99.25 (7)
C1—B5—B10—B6	−39.05 (6)	B5—B10—B12—C7	−63.48 (8)
C1—B5—B10—B9	99.95 (7)	B5—B10—B12—B8	−1.80 (9)
C1—B5—B10—B11	−0.15 (9)	B5—B10—B12—B9	36.63 (6)
C1—B5—B10—B12	63.38 (8)	B5—B10—B12—B11	−102.46 (7)
C1—B6—B10—B5	38.72 (6)	B6—C1—B3—Co2	46.53 (5)
C1—B6—B10—B9	2.24 (8)	B6—C1—B3—B4	−107.52 (7)
C1—B6—B10—B11	−98.73 (7)	B6—C1—B3—C7	−7.87 (8)
C1—B6—B10—B12	−60.63 (8)	B6—C1—B3—B8	−68.59 (8)
C1—B6—B11—Co2	−51.67 (5)	B6—C1—B4—B3	104.16 (7)
C1—B6—B11—C7	−0.05 (8)	B6—C1—B4—B5	−36.66 (7)
C1—B6—B11—B10	98.13 (7)	B6—C1—B4—B8	64.99 (8)
C1—B6—B11—B12	60.70 (8)	B6—C1—B4—B9	2.46 (9)
Co2—C1—B3—B4	−154.05 (5)	B6—C1—B5—B4	142.78 (7)
Co2—C1—B3—C7	−54.41 (5)	B6—C1—B5—B9	103.00 (7)
Co2—C1—B3—B8	−115.12 (6)	B6—C1—B5—B10	39.80 (6)
Co2—C1—B4—B3	27.83 (6)	B6—B5—B9—B4	100.60 (7)
Co2—C1—B4—B5	−112.99 (7)	B6—B5—B9—B8	63.46 (9)
Co2—C1—B4—B8	−11.35 (9)	B6—B5—B9—B10	−36.98 (6)
Co2—C1—B4—B9	−73.87 (8)	B6—B5—B9—B12	0.22 (9)
Co2—C1—B5—B4	112.15 (7)	B6—B5—B10—B9	139.00 (7)
Co2—C1—B5—B6	−30.63 (6)	B6—B5—B10—B11	38.90 (6)
Co2—C1—B5—B9	72.37 (8)	B6—B5—B10—B12	102.43 (7)
Co2—C1—B5—B10	9.17 (9)	B6—B10—B11—Co2	30.16 (6)
Co2—C1—B6—B5	151.95 (5)	B6—B10—B11—C7	98.86 (7)
Co2—C1—B6—B10	112.55 (6)	B6—B10—B11—B12	137.68 (7)
Co2—C1—B6—B11	51.02 (5)	B6—B10—B12—C7	0.31 (9)
Co2—B3—B4—C1	−26.93 (6)	B6—B10—B12—B8	61.99 (9)
Co2—B3—B4—B5	7.34 (9)	B6—B10—B12—B9	100.42 (7)
Co2—B3—B4—B8	108.35 (7)	B6—B10—B12—B11	−38.67 (7)
Co2—B3—B4—B9	70.02 (8)	B6—B11—B12—C7	−97.26 (7)
Co2—B3—C7—B8	−153.77 (5)	B6—B11—B12—B8	−64.15 (8)
Co2—B3—C7—B11	−46.51 (6)	B6—B11—B12—B9	0.03 (9)
Co2—B3—C7—B12	−116.42 (6)	B6—B11—B12—B10	37.18 (6)
Co2—B3—B8—B4	−108.13 (7)	C7—B3—B4—C1	−96.86 (6)
Co2—B3—B8—C7	27.27 (6)	C7—B3—B4—B5	−62.58 (8)
Co2—B3—B8—B9	−69.62 (8)	C7—B3—B4—B8	38.42 (6)
Co2—B3—B8—B12	−6.72 (9)	C7—B3—B4—B9	0.09 (8)
Co2—B6—B10—B5	107.28 (7)	C7—B3—B8—B4	−135.40 (7)
Co2—B6—B10—B9	70.81 (8)	C7—B3—B8—B9	−96.90 (7)
Co2—B6—B10—B11	−30.16 (6)	C7—B3—B8—B12	−33.99 (6)
Co2—B6—B10—B12	7.93 (9)	C7—B8—B9—B4	−99.95 (7)
Co2—B6—B11—C7	51.62 (5)	C7—B8—B9—B5	−62.64 (8)
Co2—B6—B11—B10	149.80 (6)	C7—B8—B9—B10	0.52 (8)
Co2—B6—B11—B12	112.37 (6)	C7—B8—B9—B12	38.55 (6)
Co2—C7—B8—B3	−28.14 (6)	C7—B8—B12—B9	−135.08 (7)
Co2—C7—B8—B4	11.27 (9)	C7—B8—B12—B10	−96.60 (7)
Co2—C7—B8—B9	74.02 (7)	C7—B8—B12—B11	−32.89 (6)
Co2—C7—B8—B12	113.11 (7)	C7—B11—B12—B8	33.11 (6)
Co2—C7—B11—B6	−50.99 (5)	C7—B11—B12—B9	97.29 (7)
Co2—C7—B11—B10	−112.37 (6)	C7—B11—B12—B10	134.44 (7)
Co2—C7—B11—B12	−151.92 (6)	B8—B3—B4—C1	−135.28 (7)
Co2—C7—B12—B8	−111.94 (7)	B8—B3—B4—B5	−101.00 (7)
Co2—C7—B12—B9	−72.23 (8)	B8—B3—B4—B9	−38.33 (6)
Co2—C7—B12—B10	−9.12 (9)	B8—B3—C7—Co2	153.77 (5)
Co2—C7—B12—B11	30.73 (6)	B8—B3—C7—B11	107.26 (7)
Co2—B11—B12—C7	−28.58 (6)	B8—B3—C7—B12	37.35 (7)
Co2—B11—B12—B8	4.53 (9)	B8—B4—B5—C1	−97.52 (7)
Co2—B11—B12—B9	68.71 (8)	B8—B4—B5—B6	−64.63 (8)
Co2—B11—B12—B10	105.86 (7)	B8—B4—B5—B9	37.42 (6)
Co2—C21—C22—C23	−60.38 (6)	B8—B4—B5—B10	−0.95 (9)
Co2—C21—C25—C24	60.51 (6)	B8—B4—B9—B5	−138.14 (7)
Co2—C22—C23—C24	−59.49 (6)	B8—B4—B9—B10	−100.39 (7)
Co2—C23—C24—C25	−58.41 (6)	B8—B4—B9—B12	−36.98 (6)
Co2—C24—C25—C21	−59.85 (6)	B8—C7—B11—Co2	115.47 (6)
B3—C1—B4—B5	−140.82 (7)	B8—C7—B11—B6	64.48 (8)
B3—C1—B4—B8	−39.18 (6)	B8—C7—B11—B10	3.10 (9)
B3—C1—B4—B9	−101.70 (7)	B8—C7—B11—B12	−36.45 (7)
B3—C1—B5—B4	37.85 (7)	B8—C7—B12—B9	39.70 (6)
B3—C1—B5—B6	−104.93 (7)	B8—C7—B12—B10	102.82 (7)
B3—C1—B5—B9	−1.93 (9)	B8—C7—B12—B11	142.67 (7)
B3—C1—B5—B10	−65.12 (8)	B8—B9—B10—B5	−100.59 (7)
B3—C1—B6—Co2	−45.97 (5)	B8—B9—B10—B6	−63.70 (8)
B3—C1—B6—B5	105.98 (7)	B8—B9—B10—B11	1.27 (9)
B3—C1—B6—B10	66.57 (8)	B8—B9—B10—B12	37.92 (6)
B3—C1—B6—B11	5.04 (8)	B8—B9—B12—C7	−38.96 (6)
B3—B4—B5—C1	−34.39 (6)	B8—B9—B12—B10	−137.34 (7)
B3—B4—B5—B6	−1.50 (9)	B8—B9—B12—B11	−100.39 (7)
B3—B4—B5—B9	100.55 (7)	B9—B4—B5—C1	−134.94 (7)
B3—B4—B5—B10	62.18 (8)	B9—B4—B5—B6	−102.05 (7)
B3—B4—B8—C7	−38.57 (6)	B9—B4—B5—B10	−38.37 (6)
B3—B4—B8—B9	−136.98 (7)	B9—B4—B8—B3	136.98 (7)
B3—B4—B8—B12	−100.00 (7)	B9—B4—B8—C7	98.42 (7)
B3—B4—B9—B5	−99.91 (7)	B9—B4—B8—B12	36.98 (6)
B3—B4—B9—B8	38.23 (6)	B9—B5—B6—C1	−97.22 (7)
B3—B4—B9—B10	−62.16 (8)	B9—B5—B6—Co2	−68.72 (8)
B3—B4—B9—B12	1.25 (9)	B9—B5—B6—B10	37.25 (6)
B3—C7—B8—B4	39.41 (6)	B9—B5—B6—B11	−0.20 (9)
B3—C7—B8—B9	102.16 (7)	B9—B5—B10—B6	−139.00 (7)
B3—C7—B8—B12	141.25 (7)	B9—B5—B10—B11	−100.10 (7)
B3—C7—B11—Co2	45.99 (6)	B9—B5—B10—B12	−36.57 (7)
B3—C7—B11—B6	−5.00 (9)	B9—B8—B12—C7	135.08 (7)
B3—C7—B11—B10	−66.38 (8)	B9—B8—B12—B10	38.49 (6)
B3—C7—B11—B12	−105.93 (7)	B9—B8—B12—B11	102.19 (7)
B3—C7—B12—B8	−37.40 (6)	B9—B10—B11—Co2	−71.24 (8)
B3—C7—B12—B9	2.31 (9)	B9—B10—B11—B6	−101.40 (7)
B3—C7—B12—B10	65.42 (8)	B9—B10—B11—C7	−2.54 (8)
B3—C7—B12—B11	105.27 (7)	B9—B10—B11—B12	36.28 (6)
B3—B8—B9—B4	−38.59 (6)	B9—B10—B12—C7	−100.11 (7)
B3—B8—B9—B5	−1.27 (9)	B9—B10—B12—B8	−38.43 (6)
B3—B8—B9—B10	61.89 (8)	B9—B10—B12—B11	−139.09 (7)
B3—B8—B9—B12	99.92 (7)	B10—B5—B6—C1	−134.47 (7)
B3—B8—B12—C7	34.05 (6)	B10—B5—B6—Co2	−105.97 (7)
B3—B8—B12—B9	−101.03 (7)	B10—B5—B6—B11	−37.45 (6)
B3—B8—B12—B10	−62.55 (8)	B10—B5—B9—B4	137.58 (7)
B3—B8—B12—B11	1.16 (9)	B10—B5—B9—B8	100.44 (7)
B4—C1—B3—Co2	154.05 (5)	B10—B5—B9—B12	37.20 (7)
B4—C1—B3—C7	99.65 (7)	B10—B6—B11—Co2	−149.80 (6)
B4—C1—B3—B8	38.93 (6)	B10—B6—B11—C7	−98.17 (7)
B4—C1—B5—B6	−142.78 (7)	B10—B6—B11—B12	−37.42 (6)
B4—C1—B5—B9	−39.78 (6)	B10—B9—B12—C7	98.38 (7)
B4—C1—B5—B10	−102.98 (7)	B10—B9—B12—B8	137.34 (7)
B4—C1—B6—Co2	−115.54 (6)	B10—B9—B12—B11	36.95 (6)
B4—C1—B6—B5	36.41 (6)	B10—B11—B12—C7	−134.44 (7)
B4—C1—B6—B10	−3.00 (9)	B10—B11—B12—B8	−101.33 (7)
B4—C1—B6—B11	−64.53 (8)	B10—B11—B12—B9	−37.15 (7)
B4—B3—C7—Co2	114.90 (6)	B11—B6—B10—B5	137.44 (7)
B4—B3—C7—B8	−38.87 (6)	B11—B6—B10—B9	100.97 (7)
B4—B3—C7—B11	68.39 (8)	B11—B6—B10—B12	38.10 (7)
B4—B3—C7—B12	−1.52 (9)	B11—C7—B8—B3	−104.49 (7)
B4—B3—B8—C7	135.40 (7)	B11—C7—B8—B4	−65.08 (8)
B4—B3—B8—B9	38.51 (6)	B11—C7—B8—B9	−2.33 (9)
B4—B3—B8—B12	101.41 (7)	B11—C7—B8—B12	36.76 (7)
B4—B5—B6—C1	−33.04 (6)	B11—C7—B12—B8	−142.67 (7)
B4—B5—B6—Co2	−4.54 (9)	B11—C7—B12—B9	−102.96 (7)
B4—B5—B6—B10	101.43 (7)	B11—C7—B12—B10	−39.85 (6)
B4—B5—B6—B11	63.98 (8)	B11—B10—B12—C7	38.98 (6)
B4—B5—B9—B8	−37.14 (6)	B11—B10—B12—B8	100.66 (7)
B4—B5—B9—B10	−137.58 (7)	B11—B10—B12—B9	139.09 (7)
B4—B5—B9—B12	−100.38 (7)	B12—C7—B8—B3	−141.25 (7)
B4—B5—B10—B6	−100.74 (7)	B12—C7—B8—B4	−101.85 (7)
B4—B5—B10—B9	38.26 (6)	B12—C7—B8—B9	−39.10 (6)
B4—B5—B10—B11	−61.84 (8)	B12—C7—B11—Co2	151.92 (6)
B4—B5—B10—B12	1.69 (9)	B12—C7—B11—B6	100.93 (7)
B4—B8—B9—B5	37.31 (6)	B12—C7—B11—B10	39.55 (6)
B4—B8—B9—B10	100.47 (7)	B12—B8—B9—B4	−138.51 (7)
B4—B8—B9—B12	138.51 (7)	B12—B8—B9—B5	−101.19 (8)
B4—B8—B12—C7	97.83 (7)	B12—B8—B9—B10	−38.03 (6)
B4—B8—B12—B9	−37.25 (6)	B12—B9—B10—B5	−138.51 (7)
B4—B8—B12—B10	1.23 (9)	B12—B9—B10—B6	−101.62 (7)
B4—B8—B12—B11	64.94 (8)	B12—B9—B10—B11	−36.64 (6)
B4—B9—B10—B5	−37.78 (6)	B12—B10—B11—Co2	−107.52 (7)
B4—B9—B10—B6	−0.89 (9)	B12—B10—B11—B6	−137.68 (7)
B4—B9—B10—B11	64.08 (8)	B12—B10—B11—C7	−38.82 (6)
B4—B9—B10—B12	100.73 (7)	C21—C22—C23—Co2	59.97 (6)
B4—B9—B12—C7	−2.11 (9)	C21—C22—C23—C24	0.48 (10)
B4—B9—B12—B8	36.85 (6)	C22—C21—C25—Co2	−59.46 (7)
B4—B9—B12—B10	−100.49 (7)	C22—C21—C25—C24	1.05 (10)
B4—B9—B12—B11	−63.54 (8)	C22—C23—C24—Co2	58.58 (6)
B5—C1—B3—Co2	116.26 (6)	C22—C23—C24—C25	0.17 (10)
B5—C1—B3—B4	−37.79 (7)	C23—C24—C25—Co2	59.09 (6)
B5—C1—B3—C7	61.85 (8)	C23—C24—C25—C21	−0.76 (10)
B5—C1—B3—B8	1.13 (9)	C25—C21—C22—Co2	59.44 (6)
B5—C1—B4—B3	140.82 (7)	C25—C21—C22—C23	−0.94 (10)
B5—C1—B4—B8	101.65 (7)		
