# Supplementary figures and images for: Crystal structure of a second polymorph of 2-cyclo­penta­dienyl-1,7-dicarba-2-cobalta-closo-dodeca­borane(11)
Source: Acta Crystallogr E Crystallogr Commun. 2015 Jun 17;71(Pt 7):m141–2. doi: 10.1107/S2056989015011445 (PMC4518921; doi:10.1107/S2056989015011445)

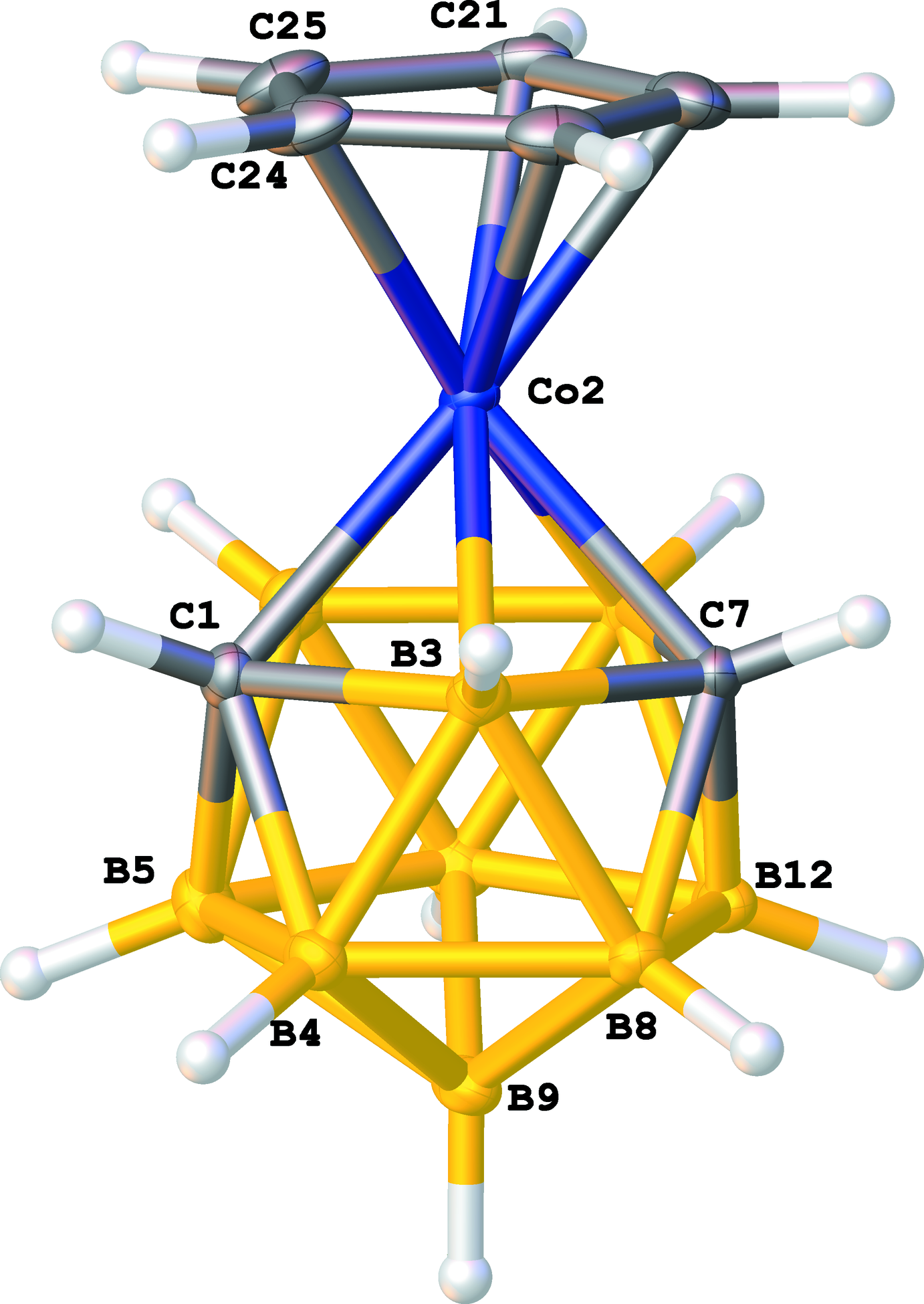

Supplement: Supplementary file 4 [file e-71-0m141-fig1.tif]

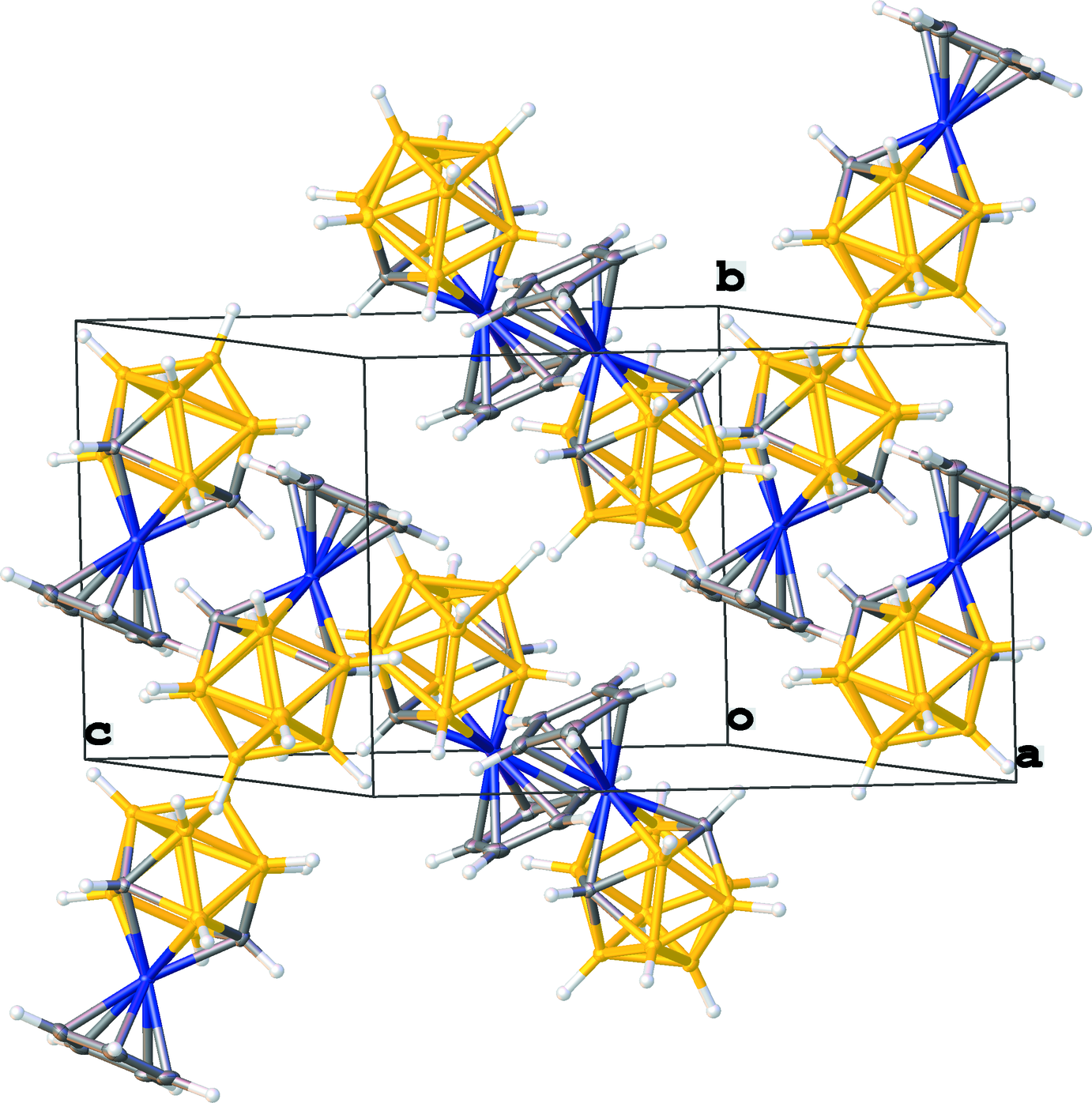

Supplement: Supplementary file 5 [file e-71-0m141-fig2.tif]
